# Layered MoS_2_: effective and environment-friendly nanomaterial for photocatalytic degradation of methylene blue

**DOI:** 10.1038/s41598-023-41279-y

**Published:** 2023-08-29

**Authors:** Joanna Kisała, Renata Wojnarowska-Nowak, Yaroslav Bobitski

**Affiliations:** 1 Institute of Biology, University of Rzeszow, Pigonia 1 Str., 35-310 Rzeszow, Poland; 2 Institute of Materials Science, College of Natural Sciences, University of Rzeszow, Pigonia 1 Str., 35-959 Rzeszow, Poland; 3 Centre for Microelectronics and Nanotechnology, Institute of Physics, University of Rzeszow, Pigonia 1, 35-959 Rzeszow, Poland

**Keywords:** Environmental sciences, Chemistry, Materials science, Nanoscience and technology

## Abstract

Photocatalytic degradation is a promising method for removing persistent organic pollutants from water because of its low cost (see solar-driven photocatalysis), high mineralisation of pollutants, and low environmental impact. Photocatalysts based on transition metal dichalcogenides (TMDs) have recently attracting high scientific interest due to their unique electrical, mechanical, and optical properties. A MoS_2_ photocatalyst of the layered structure was managed to photodegrade methylene blue (MB) under visible light irradiation. The catalyst was thoroughly characterised using SEM, AFM, powder XRD, UV–Vis, Raman, and XPS measurements. The photocatalytic degradation of the MB solution was conducted under the following conditions: (i) reductive and (ii) oxidative. The impact of optical and electronic properties, and the MoS_2_-MB interaction on photocatalytic activity, was discussed. The apparent rate constants (k_app_) of degradation were 3.7 × 10^–3^; 7.7 × 10^–3^; 81.7 × 10^–3^ min^−1^ for photolysis, oxidative photocatalysis, and reductive photocatalysis. Comparison of the degradation efficiency of MB in reductive and oxidative processes indicates the important role of the reaction with the surface electron. In the oxidation process, oxygen reacts with an electron to form a superoxide anion radical involved in further transformations of the dye, whereas, in the reduction process, the addition of an electron destabilises the chromophore ring and leads to its rupture.

## Introduction

Human activity causes water pollution with chemicals produced during various technological processes. The increasing use of chemicals is due to the current way of life and the continuous growth of the population. This is projected to put more pressure on natural ecosystems and human populations in the near future. Therefore, the development of new wastewater treatment technologies is an important environmental issue. In recent decades, much attention has been devoted to the development of new wastewater treatment methods^1^. Among them, heterogeneous photocatalysis seems to be very promising, but the most effective catalysts are based on expensive and rare precious metals such as platinum and gold. Recent research^2,3^ indicates that transition metal dichalcogenides may be a cheap and handy alternative to precious metal catalysts.

Molybdenite (MoS_2_) scores in the family of transition metal dichalcogenides (TMDs). Molybdenum disulfide is a layered material with sandwich-like structure that reveals many unique properties^4^. Its features make it possible to use it as a photocatalyst. MoS_2_ exists in three phases, two stable semiconductor phases with a trigonal prismatic structure (2H and 3R) and a metallic octahedral metastable phase (1T). Bulk MoS_2_ has an indirect band gap of ~ 1.2 eV, which changes to a direct band gap of ~ 1.9 eV after the reduction of layers^5^. It indicates that the material has a strong absorption effect on the sunlight. MoS_2_ nanomaterials provide good catalytic activity due to the high absorption response in the visible wavelength range. The disadvantage of this material is the rapid recombination of photogenerated electron–hole pairs^6^. Charge separation can be improved by increasing the ratio of the metal edge sites (plain of the edges 100) to the face (basal plane 002). The active centers of the catalyst are mainly concentrated at the edge sites and S vacancies, and its basal plane is considered chemically inert. The edge of the crystal has a high surface energy, which makes MoS_2_ able to react quickly with oxygen. In addition, single-layer MoS_2_ has excellent charge carrier mobility, as good as that of carbon nanotubes^7^.

Considerable efforts have been made to investigate the catalytic activity of various MoS_2_ nanostructures, such as nanoparticles, mesopores, nanowires, amorphous MoS_2_, thin films and chemically exfoliated MoS_2_ layers. MoS_2_ has been used in environmental engineering for the treatment of organic pollutants by both adsorption and photocatalytic degradation^8–14^.

Photocatalytic properties of pristine MoS_2_ were tested in photocatalytic dyes degradation by several research groups. Lin et al.^15^ have synthesised monolayered MoS_2_ nanocrystals (NCs) and S-depleted MoS_1.65_ NCs. The solar-driven photocatalysis of the MB solution was investigated. The photodegradation of MB without NCs reached about 50% after 440 min, whereas the presence of MoS_2_ NCs and S-depleted MoS_1.65_ resulted in complete MB photodegradation after 180 min. and 7 min., respectively. S-depleted NCs also showed excellent photocatalytic efficiency in methyl orange (MO) degradation. It was found that MO was fully degraded within 3 min. The authors not appointed the value of the degradation reaction rate constant. Karmar et al.^16^ hydrothermally have obtained MoS_2_ nanoplatelets (MNPs) (S1), a few-layer MoS_2_ nanosheets (MNSs) (S2) and nanorods (MNRs) (S1#800C). The photocatalytic properties of these materials were evaluated in MB degradation. The obtained reaction rate constants were 8.1 × 10^–3^ min^−1^, 5.7 × 10^–3^ min^−1^, and 10.0 × 10^–3^ min^−1^ for S1, S1#800C, and S2, respectively. Chandhary et al.^17^ compared photocatalytic performance between bulk MoS_2_ and nanosheets by degrading MB aqueous solution under sunlight irradiation. The degradation rate constant for MoS_2_ nanosheets was 27.6 × 10^–3^ min^−1^ and for bulk MoS_2_ 3.5 × 10^–3^ min^−1^. Lai et al.^18^ have received highly expanded interlayer spaces MoS_2_ photocatalyst in the presence of Pluronic F-127 as a template at different pH (1, 3, and 5 denoted as MF-1, MF-3, and MF-5, respectively). MoS_2_ synthesised without of template, and with template without pH adjusting were assigned as M and MF, respectively. The highly expanded MoS_2_ exhibited high photocatalytic performance. The photodegradation activity of these materials decreases in order: MF-1 (k = 26.2 × 10^–3^ min^−1^) > MF-3 (k = 18.5 × 10^–3^ min^−1^) > MF (k = 10.8 × 10^–3^ min^−1^) > M (k = 9.4 × 10^–3^ min^−1^) > MF-5 (k = 8.2 × 10^–3^ min^−1^). Kisala et al. in their recent work^19^ have produced a layered MoS_2_ and assessed its photocatalytic properties in the degradation reaction of bromophenol blue (BPB) dye in weakly acidic aqueous solution (pH 5.2) in the presence of *t*-BuOH and continuous argon flow. The apparent rate constant of the dye decay was 103.7 × 10^–3^ min^−1^. The photocatalytic degradation experiment carried out by Kisała et al.^19^ differed from the other cited works in the conditions of the degradation reaction. In this article, the decomposition of the dye (BPB) occurred as a result of a reaction with an electron. However, in the remaining works, the authors did not specify the reaction conditions (e.g. access to air, which can be presumed) or the pH of the reaction mixture.

Dyes are widely used in different industries, such as textiles, food, rubber, printing, medicine, plastic, concrete, and paper industry. Common use of dyes generates a large amount of hazardous wastewater. The industry that consumes most dyes is the textile industry; one of the most widespread dyes is methylene blue (MB). Methylene blue is a cationic dye that is highly resistant to light, water, chemicals, detergents and microbial activities^20^.

Our goal was to investigate the MoS_2_ nanomaterial as a suitable photocatalyst for the degradation of MB under the influence of visible radiation. MB degradation was carried out in two systems: (i) in the presence of oxygen, an oxidative process, and (ii) under argon, in the presence of a hydroxyl radical scavenger, a reductive process. An attempt was also made to determine the MB degradation pathway in an aqueous solution.

## Methods

### Materials

Methylene blue (WARCHEM, Warsaw, Poland), *t*-BuOH (*tert*-butanol—hydroxyl radical scavenger) and the remaining reagents were of analytical grade and were used as received. The hydrogen ion concentration of the investigated systems was adjusted by NaOH and HCl. MoS_2_ was synthesised by Kisala et al.^19^.

### Catalyst characterisation

The morphology and particle size of the prepared nanoparticles were evaluated by a Helios NanoLab 650 field emission scanning electron microscope (FESEM) (FEI, Hillsboro, Oregon, USA) operating at 5 kV and 18 kV using an ETD detector with secondary electron (SE) imaging mode. Atomic force microscopy (AFM) measurements were performed using a Solver Nano II microscope (NT-MDT Spectrum Instruments LLC, Tempe, Arizona, USA). Phase identification was performed with an X-ray diffractometer (D8 Advance, Bruker, Germany) and 1.5406 Å Cu K_α_ radiation. The average crystallite size was calculated based on line broadening analysis. The XPS was performed on a KRATOS XSAM800 spectrometer (Kratos Analytical Ltd, Manchester, UK) with an excitation source of A1 Kα (hν = 1486.6 eV). Raman spectra were obtained using an inVia Micro Raman Renishaw spectrometer combined with a Leica DM 2500 M microscope (Renishaw, Wotton-under-Edge, UK) equipped with a 633 nm laser as an excitation source. UV–Vis spectra were recorded using an Agilent Technologies Cary Series UV–Vis-NIR spectrophototometer in the wavelength range of 180–1200 nm. The hydrodynamic diameter of the semiconductor particles and the electrokinetic potential (zeta potential, ζ-potential) were measured by the NanoPlus 3 HD analyser (Particulate Systems, Micromeritics, Norcross, GA 30093, USA).

### Photocatalytic experiment conditions

The MB degradation reaction was performed on MoS_2_ as a photocatalyst in the Heraeus LRS2 photoreactor (of 250 cm^3^ volume). For this purpose, 250 cm^3^ of 5.0 × 10^–4^ mol dm^−3^ MB solution containing 0.3 mol dm^−3^ *t*-BuOH (only in reductive experiment) and 0.05 g of MoS_2_ were placed in the photoreactor (mixture pH = 4.6). The resulting mixture was stirred for 30 min in the dark under argon conditions (reductive experiment) or open to air (oxidative experiment). The illumination was carried out using the excimer lamp TQ150 (150 W, with forced water cooling down to 25 °C, of power density 4.7 mW cm^−2^ measured by digital lux meter Peak Tech 5025, which gives light intensity ca. 7.9 × 10^19^ photons per second) immersed in the continuously stirred reaction suspension. The photocatalytic reaction was performed up to 120 min illumination time. During the reaction, 2 cm^3^ samples were collected from the reactor at regular time intervals.

UV–Vis spectra of organic compounds solutions were measured on a VWR UV–VIS 3100 PC spectrophotometer.

### Kinetic simulations

Competitive kinetic simulations were performed with the Kinetiscope™ stochastic kinetic simulation software^21^ freely available on the authors' web page: http://www.hinsberg.net/kinetiscope/. The rate constants of the radical reactions describing the investigated process, required for the competitive kinetics simulation, were taken from the compilation of the rate constants^22^ and shown in Supplementary Table S1.

## Results and discussion

### Catalyst characterisation

**Figure 1:**
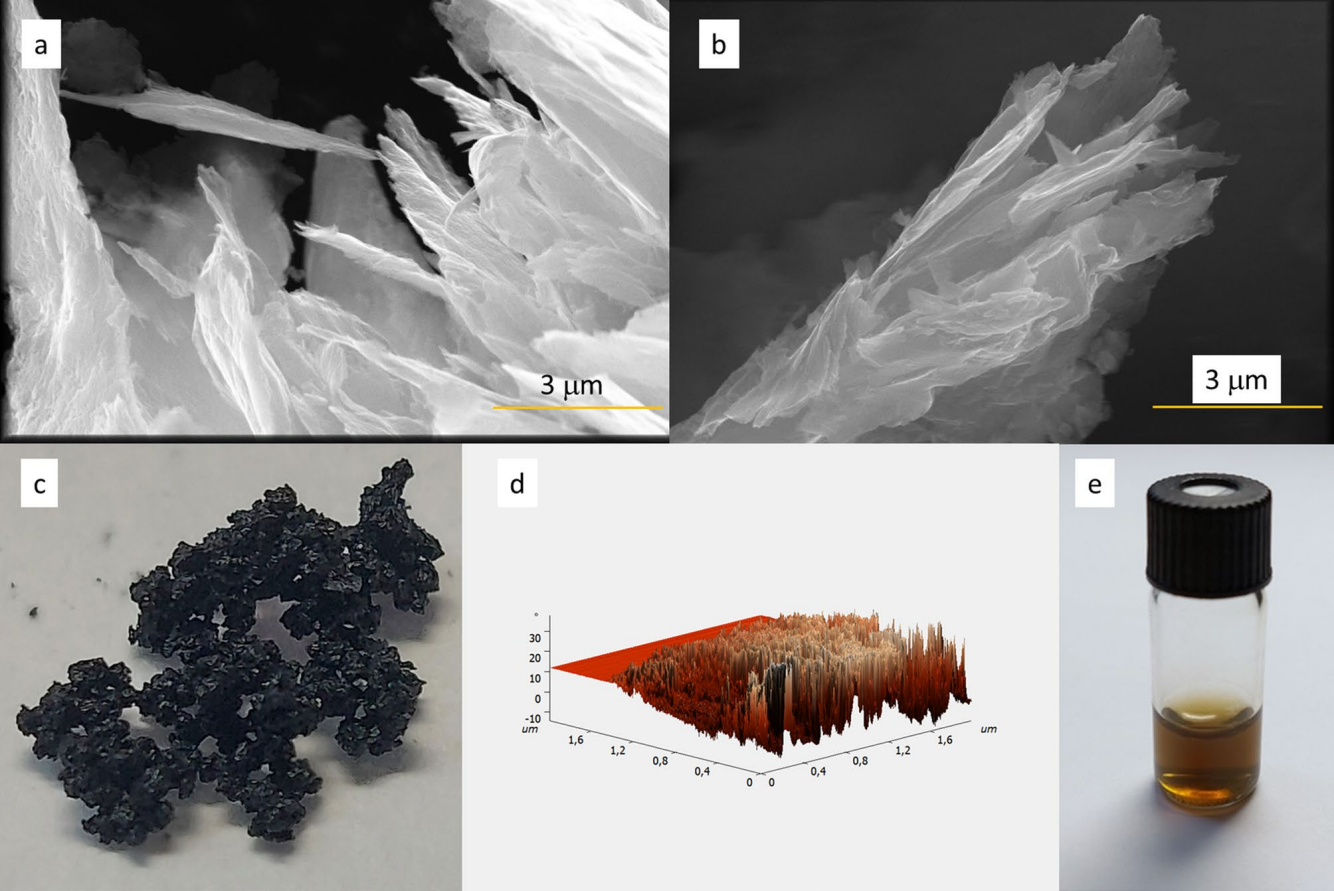
Microscale morphology (SEM) of MoS_2_ at different places (**a**,**b**) macroscale morphology of MoS_2_ (**c**), AFM height map (**d**), water suspension of MoS_2_ (**e**).

Figure 1a,b display the morphology of MoS_2_ as examined by SEM. The images demonstrate the layered structure of the material, which was beneficial for exposing more edges. The macroscopic view of the material is presented in Fig. 1c. The local morphology of the sample was observed by atomic force microscopy (Fig. 1d). The surface topography observed for MoS_2_ was rough. This suggests that the sample comprises multiple sheet-like structures, creating layered objects. A water dispersion of MoS_2_ is depicted in Fig. 1e.

**Figure 2:**
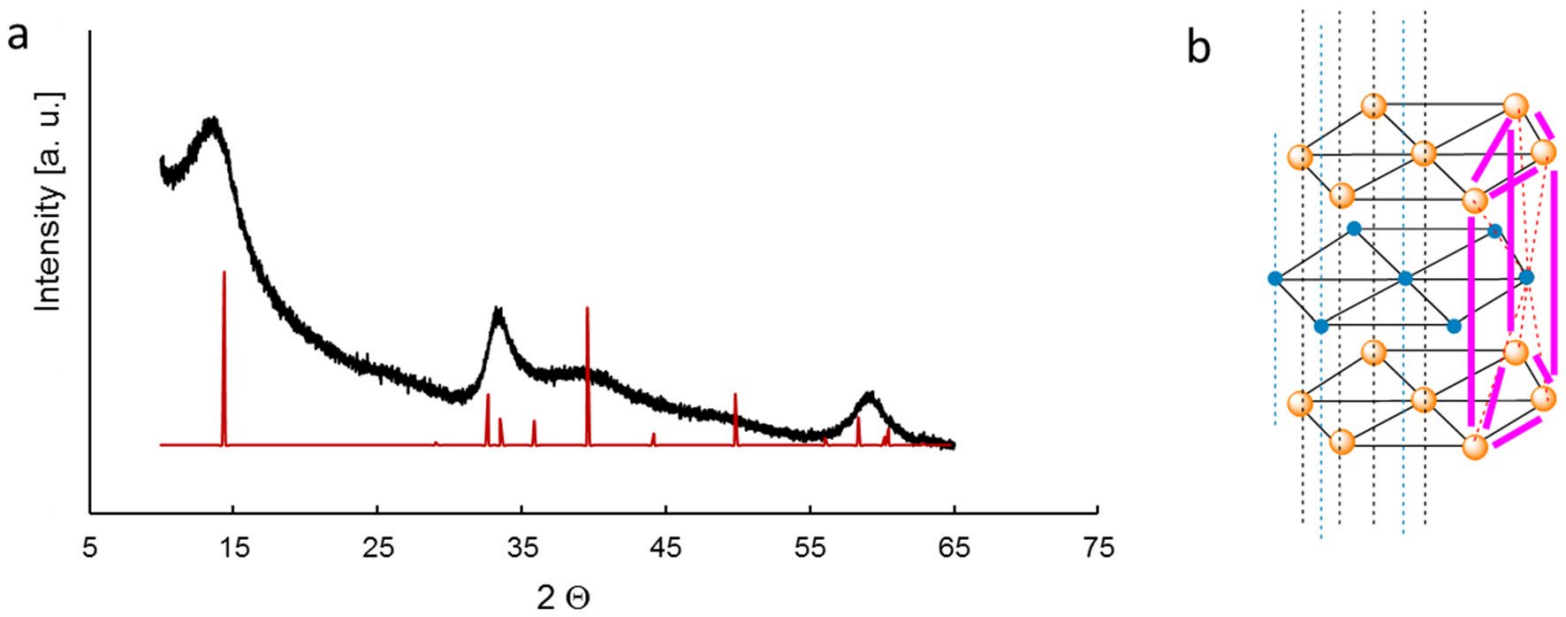
XRD pattern of MoS_2_ (examined sample—black, reference—red) (**a**), Crystal structure of 2H-MoS_2_ (**b**).

The XRD spectra (Fig. 2a) of MoS_2_ showed five distinctive 2H-MoS_2_ peaks (PDF card #01-071-9809, P63/mmc)^23^, which can be assigned to the plane (002), (100), (103), (105) and (110) of MoS2 at 2Θ values: 13.95; 33.3; 39.8; 48.2; 59.2, respectively. The crystal structure of 2H-MoS_2_ is shown in Fig. 2b. Strong intenseness of the peak at 13.95 2Θ reveal that the material has many crystal planes orientated in that direction. The peak broadening indicates a sheet-like crystallite morphology with an average size of 50 nm. Thin-layered MoS_2_ generally displays n-type behaviour. The XRD results represented that the obtained materials have a pure phase. A more thorough discussion of the XRD measurement results of this material can be found in article Kisala et al.^19^.

**Figure 3:**
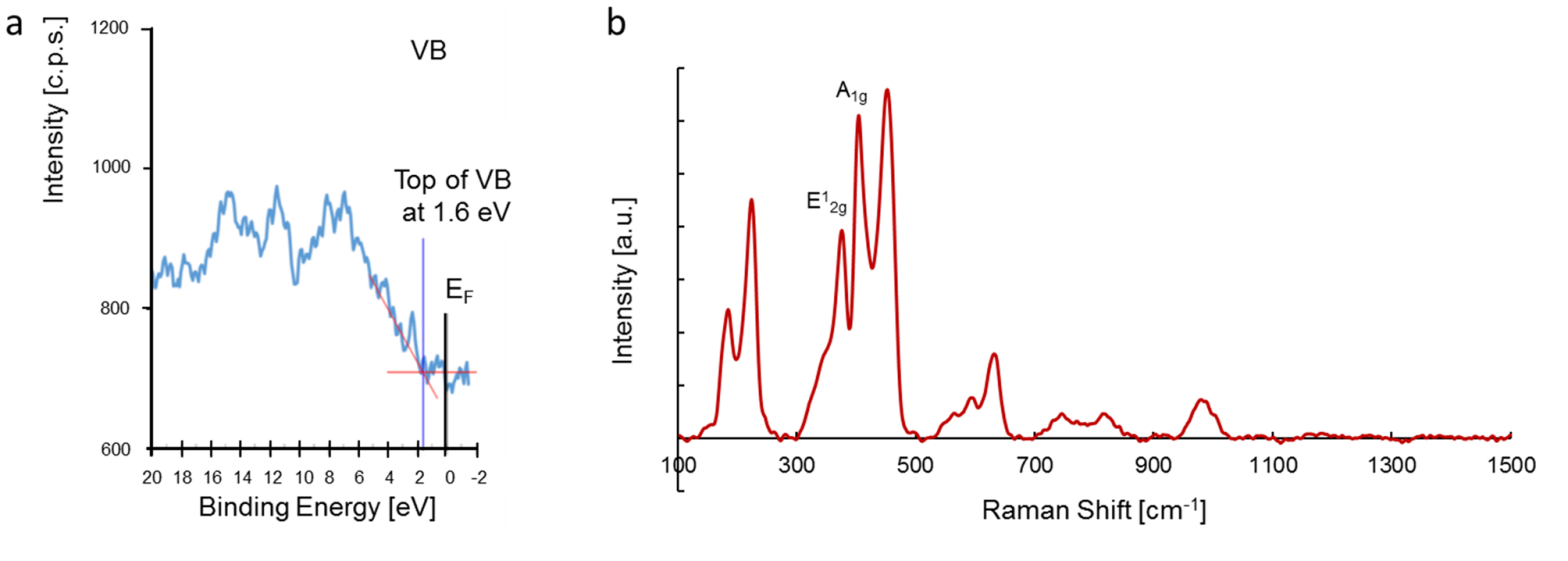
XPS Valence band energy (**a**), Raman spectra of MoS_2_ (**b**).

The XPS spectrum was measured to derive the composition of the material. From the XPS spectra (Supplementary Fig. S1), the binding energy values were 162.6 eV for S (2p) and 229.6 eV for Mo (3d)^24^. Other signals observed may be caused by partial oxidation of the material during the annealing process. The quantitative analysis gave the Mo:S ratio of 1:2.03, corresponding to the stoichiometry of MoS_2_. The valence band energy was also determined from XPS measurements as 1.6 eV (Fig. 3a). The XPS results confirmed the behaviour of MoS_2_ semiconductors as n-type.

The Raman spectrum is like a chemical fingerprint that identifies a molecule or material. In Fig. 3b, one can observe peaks at 378, 404, 451 cm^−1^, which confirmed the 2H-MoS_2_ phase^25^. The peak at 378 cm^−1^ corresponds to E_2g_^1^ and the peak at 404 cm^−1^ is assigned to A_g_^1^. These results confirm the structure of the 2H-MoS_2_ catalyst.

The optical properties of MoS_2_ were analysed by UV–Vis diffuse reflectance spectra (Supplementary Fig. S2). The material absorbed light from the ultraviolet and visible regions, which was attributed to its narrow bad gap. The absorption spectrum of MoS_2_ reflects the band gap of 2.03 eV, but it also manifests a fine structure with narrow absorption peaks at 1.9 eV (653 nm) and 2.1 eV (590 nm) associated with direct transitions from the spin–orbit split valence band to the conduction bands at the K point in the Brillouin zone.

The absorption of visible light creates consequently holes in the valence band and electrons in the conduction band without affecting essential chemical bonds in the sulphide surface.

**Figure 4:**
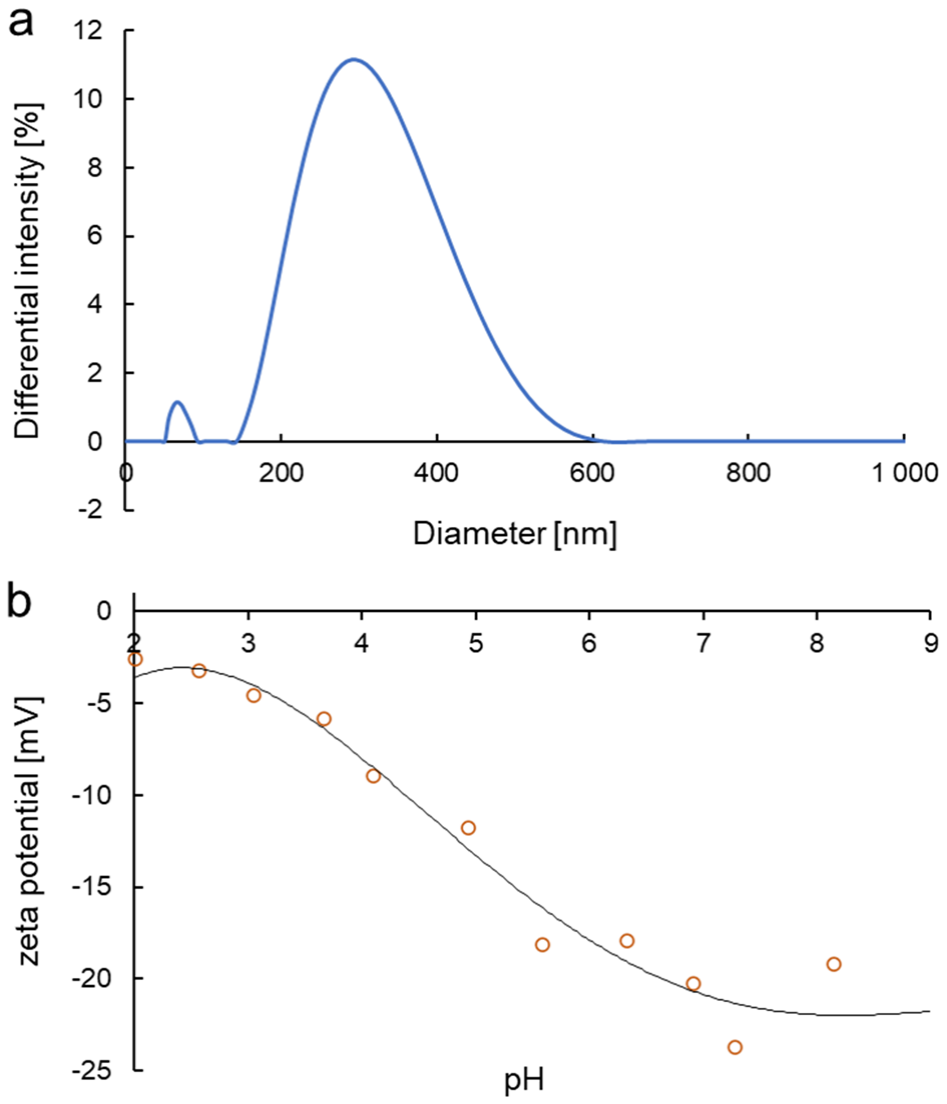
The particle size distribution of the H_2_O dispersion, obtained by DLS (**a**); zeta potential of 2D-MoS_2_ as a function of pH (**b**).

The properties of MoS_2_ in aqueous solution, surface charge, and particle size were analysed. The particle size of MoS_2_ in water was evaluated by DLS (considering its hydrodynamic radius) where platelet sizes ranged from 72 to 564 nm (Fig. 4a). The zeta potential dictates the sign and amount of surface charge in relation to the surrounding conditions. The ζ-potential of MoS_2_ was negative in all pH range (2–9) (Fig. 4b). The results showed a decrease of the zeta potential of the particles with an increase of the pH value. The face/edge ratio strongly influences the magnitude of zeta potential; with increasing this ratio, the magnitude decreases^19^.

### Photocatalytic properties

**Figure 5:**
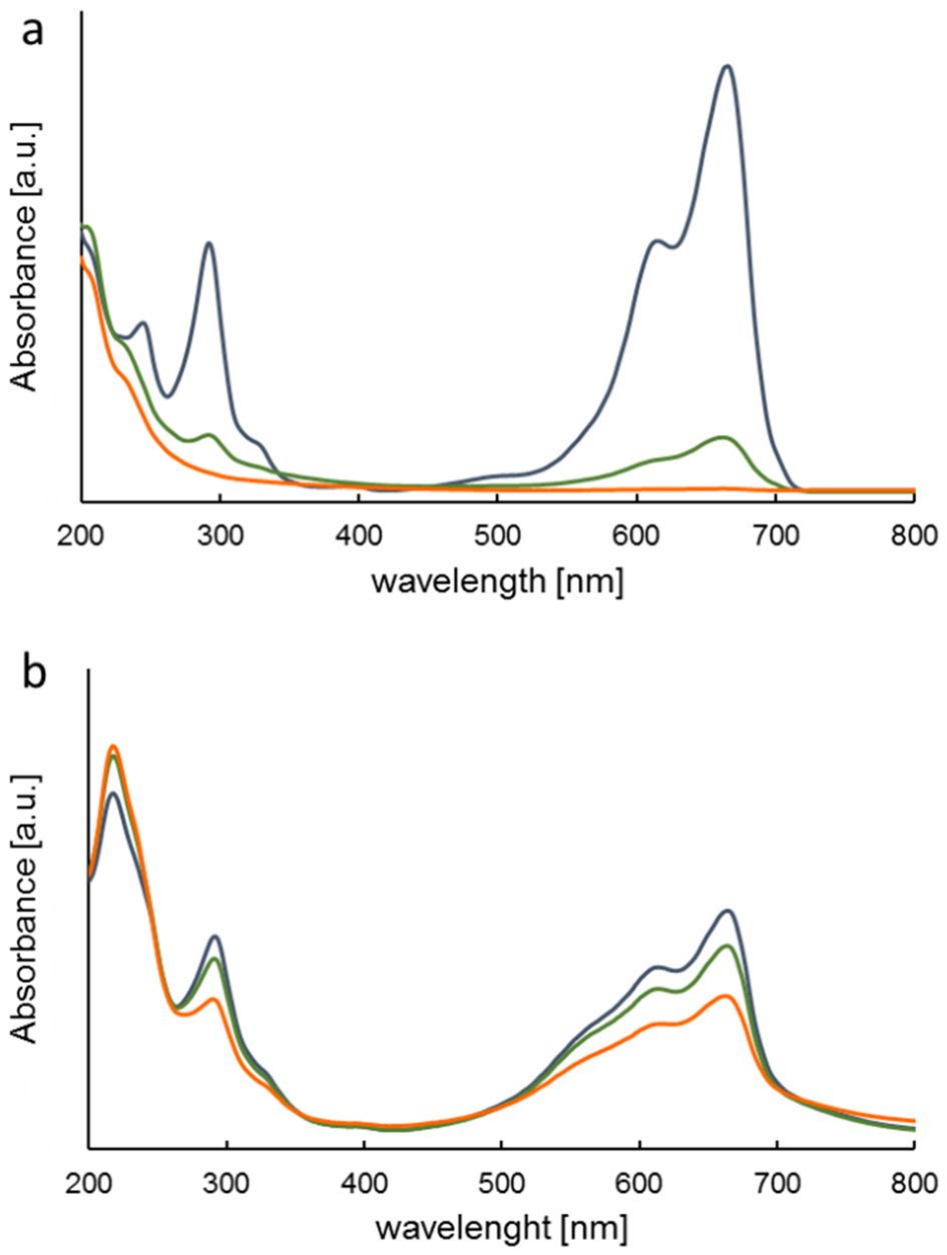
UV–Vis spectrum changes of MB in time under reductive conditions (**a**), oxidative conditions (**b**); irradiation time 0 min—dark blue, 20 min—green, 120 min—orange.

The photocatalytic properties of the MoS_2_ material were investigated by monitoring the photocatalytic degradation of the MB solution under different conditions: (i) reductive and (ii) oxidative. Figure 5 presents changes in UV–Vis spectra of MB in time.

The progress of the degradation reaction was monitored by measuring the changes in MB concentration in specified time intervals. The photocatalytic degradation of MB on MoS_2_ was performed at pH 4.6. The kinetics of MB degradation follow a pseudo-first-order model Eq. (1):

1 $$\mathrm{ln}\frac{{C}_{t}}{{C}_{0}}=- {k}_{app}t,$$

where k_app_ is the apparent rate constant; C_0_ and C_t_ are the initial concentration and concentration at time t.

**Figure 6:**
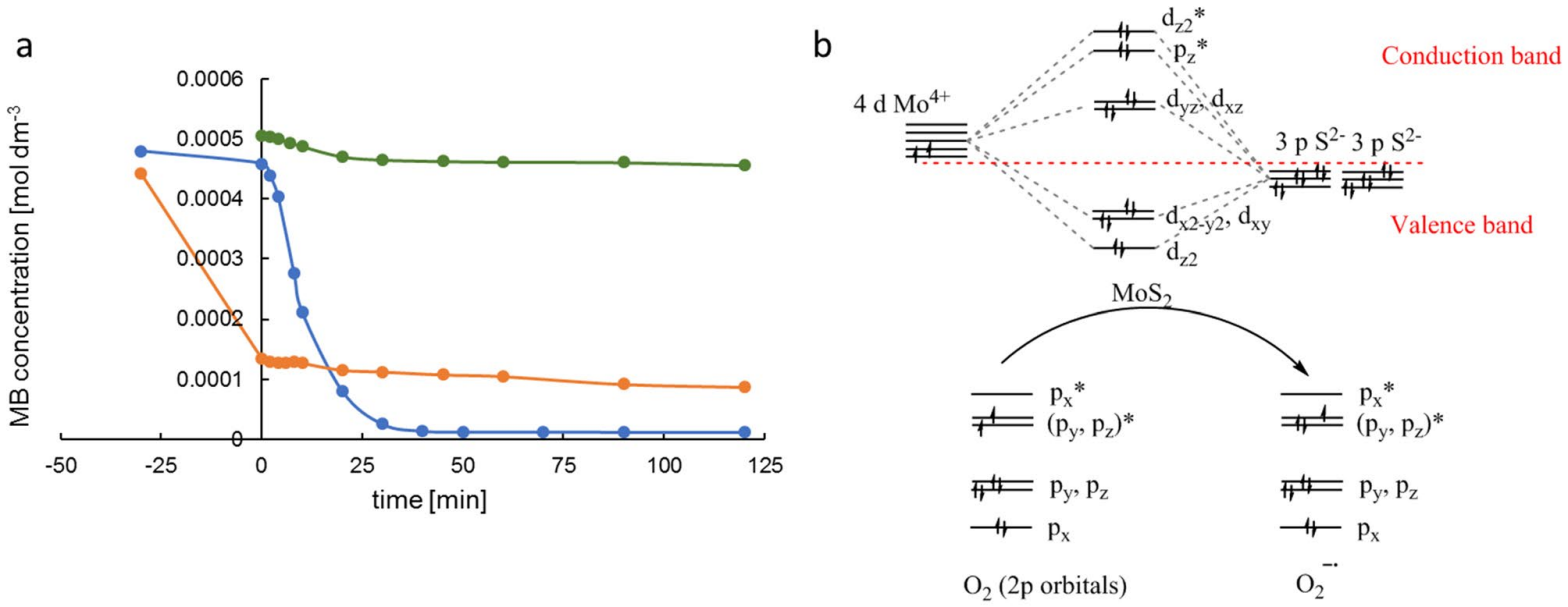
Disappearance of MB in oxidative photocatalysis (red), reductive photocatalysis (blue), and photolysis (green) (**a**); molecular orbital energy level diagram for Mo(S)_6_ in 2H-MoS_2_ and O_2_/O_2_^**⋅**−^ (**b**).

A comparison of MB concentration changes in time for photolysis, oxidative photocatalysis, and reductive photocatalysis indicates reductive photocatalysis is the most efficient in MB degradation (Fig. 6a). However, the degradation efficiency calculated from Eq. (2) in both photocatalysis cases is high (97.5%, 80.3% for the reductive and oxidative processes, respectively). The high efficiency of degradation in the oxidative process is mainly caused by processes that take place in the dark period.

2 $$\%{D}_{eff}= \left(\frac{{C}_{-30}-{C}_{120}}{{C}_{120}}\right)\cdot 100\%,$$

where C_− 30_ is the concentration of MB in time – 30 min; C_120_ is the concentration of MB in time 120 min.

The kinetics of MB disappearance represented in Fig. 6a, with an adsorption period in the dark of 30 min, show significant differences between oxidative and reductive processes in the dark period. The MB concentration rapidly decreased in the oxidative process, whereas in the reductive process, one can observe only adsorption on the catalyst surface. We postulate that the decrease of MB concentration in the oxidative process is associated with the presence of oxygen in the reaction mixture. After starting illumination, the reaction enters the slow deceleration stage, in which the concentration of MB slowly decreased. The observed changes are caused by the rapid generation of superoxide radicals in the dark process. The oxygen dissolved in the reaction mixture is adsorbed on the face of MoS_2_ (the face has a hydrophobic nature) and radicals are generated. The O_2_^**⋅**−^ generation rate falls rapidly over time and the reaction slows down. It must be caused by the rapid consumption of oxygen in the reaction mixture. The solubility of oxygen in distilled water is 2.83 × 10^–4^ mol dm^−3^^26^.

The electronic structure of MoS_2_ is determined by the ligand field splitting of the Mo 4d states in the field of the S^2−^ anions since the D_*6h*_^4^ space group of the trigonal layers contains a unique z-axis, the S 3p states subdivide into two groups (p_z_; p_x_ and p_y_), and the Mo 4d into three groups (d_z2_; d_xy_ and d_x2–y2_; d_xz_ and d_yz_) (Fig. 6b). The upper part of the valence band is determined by the d orbitals of Mo (d_x2–y2_; d_xz)_ and the p orbitals of sulphur (p_x_ and p_y_). The band of the antibonding Mo–d–S–p states is located above. The consideration of the 6 Mo valence electrons and a formal charge of − 2 for sulphur give a formal charge of + 4 for Mo in MoS_2_. The dz^2^ band is fully occupied, which easily explains the semiconducting behaviour of MoS_2_. Figure 6b depicts the probable interaction of MoS_2_ with the oxygen molecule.

The distribution of MB ionic species is presented in Supplementary Fig. S3. The MB solution at pH = 4.6 consists of: [MB]^+^  = 0.958; [MBH]^2+^ = 0.042^27^. Therefore, the main entity that participates in the reactions is the cation [MB]^+^. The efficiency of catalysis depends on the type of catalyst-substrate interactions. The negative zeta potential of the catalyst suggests the presence of anions in the Stern layer. Methylene blue is a cationic dye that is positively charged under the reaction conditions, hence the driving force of the catalysis is the interaction of a positively charged dye and a negatively charged catalyst surface. It allows the transfer of electrons from the surface substrate to the MB molecule.

**Scheme 1:**
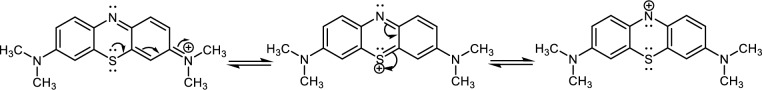
Resonance structures of MB cation.

Scheme 1 presents classical Lewis formulae, but it should be noted that the formal positive charge of the dye is not placed on the sulphur atom but is distributed over a wide delocalised range outside the thiazine ring. Terminal methyl groups attached to nitrogen atoms bear the most positive charges of the cation^28^.

**Figure 7:**
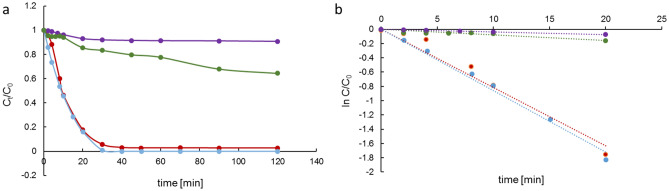
MB degradation in time in photolysis (violet), oxidative photocatalysis (green), reductive photocatalysis (red), simulations of reductive photocatalysis (light blue) (**a**); First order linear transforms of MB concentrations changes in time in photolysis (violet), oxidative photocatalysis (green), reductive photocatalysis (red), simulations of reductive photocatalysis (light blue) (**b**).

Figure 7a shows the time dependent decomposition of MB dye under light irradiation. A linear dependence was obtained between ln (C_t_/C_0_) and the irradiation time (Fig. 7b), which indicates that reactions follow the pseudo-first-order mechanism. The apparent rate constants (k_app_) of degradation were 3.7 × 10^–3^; 7.7 × 10^–3^; 81.7 × 10^–3^; 86.1 × 10^–3^ min^−1^ for photolysis, oxidative photocatalysis, reductive photocatalysis, and simulation for reductive photocatalysis. The k_app_ obtained for simulation is in good agreement with the reductive process.

The irradiation of MoS_2_ causes its band-gap excitation and the generation of valence-band holes and conduction-band electrons. The observed loss of dye concentration occurs mainly as a result of the reaction with e^–^ and h^+^ present on the surface of the catalyst. Continuous blowing of the reaction mixture with argon was used in the reduction process to prevent access to oxygen, and *t*-BuOH was used as a scavenger of holes, and hydrogen atoms that can be formed in a reductive experiment (Eqs. 3–4)^29^. Such conditions mean that the mobility of electrons plays a major role in the degradation process. The layered structure of MoS_2_ favours the separation of charge carriers. The presence of positive and negative charge densities on the Mo-edges and S-faces induces a polarisation effect on these nanoparticles, which effectively separates the electron–hole pair.

3 $${\left(C{H}_{3}\right)}_{3}COH+H \to {}^{\cdot }C{H}_{2}{\left(C{H}_{3}\right)}_{2}COH+ {H}_{2},$$

4 $${\left(C{H}_{3}\right)}_{3}COH+{h}^{+} \to {}^{\cdot }C{H}_{2}{\left(C{H}_{3}\right)}_{2}COH+ {H}^{+}.$$

**Figure 8:**
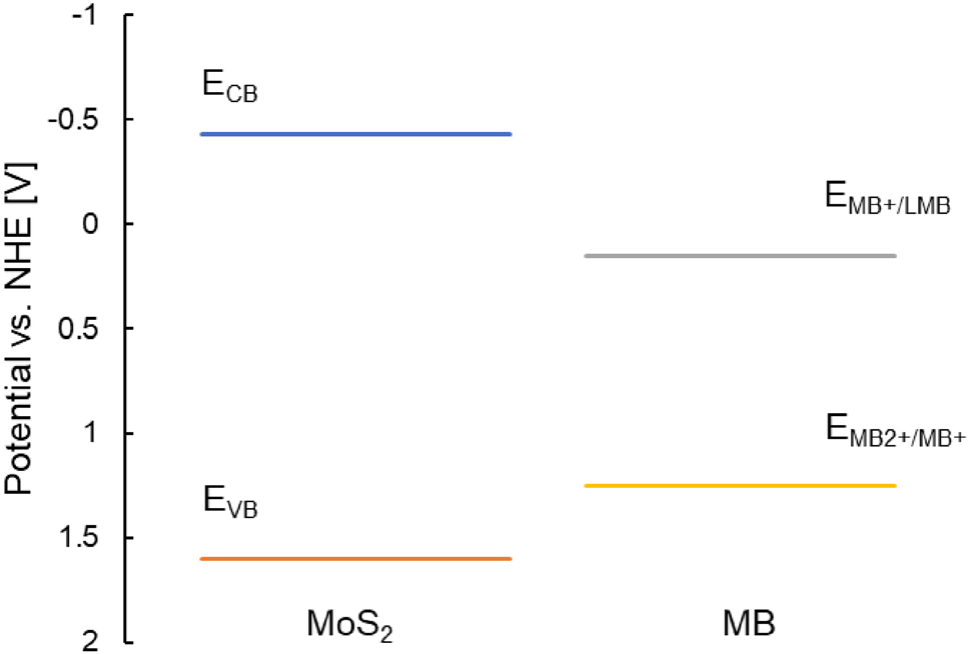
Diagram of the electrochemical potentials of the conduction and valence MoS_2_ bands, and the oxidation^31^ and reduction^33^ of MB dye.

Note that a water molecule or hydroxyl anion is not oxidised at the hole to form a hydroxyl radical (^**⋅**^OH) because the MoS_2_ valence band potential (E_VB_ =  + 1.6 V) is less positive than the oxidation potential of the hydroxyl ion (E_**⋅**OH/−OH_ = + 1.9 V), or water molecules (E_**⋅**OH,H+/H2O_ =+ 2.73 V)^30^. However, the hole could contribute to MB degradation due to its redox potential (E_MB2+/MB+_ =  + 1.25 V, Fig. 8)^31^.

As a result of superoxide radical post-reactions, hydroxyl radicals may be formed^32^:

5 $${O}_{2}+{e}^{-}\to {O}_{2}^{\cdot -},$$

6 $${O}_{2}^{\cdot -}+ {H}^{+}\to {HOO}^{\cdot },$$

7 $${HOO}^{\cdot }+{e}^{-}\to {HOO}^{-},$$

8 $${HOO}^{-}+{H}^{+}\to HOOH,$$

9 $$HOOH+ {e}^{-} \to {HO}^{\cdot }+ {HO}^{-}.$$

The Eqs. (6)–(9) may be summarised as Eq. (10). Formed in reaction (9) hydrogen peroxide is the source of hydroxyl radicals (Eq. 11).

10 $${O}_{2}+2{e}^{-}+2{H}^{+}\to {H}_{2}{O}_{2},$$

11 $${{H}_{2}O}_{2}+{e}^{-}\to {}^{\cdot }OH+ {}^{-}OH.$$

The rate of the reaction of O_2_^**⋅**−^ with a proton is diffusion controlled and is 5 × 10^10^ mol^−1^ s^−1^. The protonation rate of O_2_^**⋅**−^ by proton donors (e.g. MB) is much smaller and depends on the ease of detaching the hydrogen atom from the molecule. Hence, ROS such as O_2_^**⋅**−^, ^**⋅**^OH, H_2_O_2_, ^**⋅**^OOH may take part in the oxidative process.

**Scheme 2:**
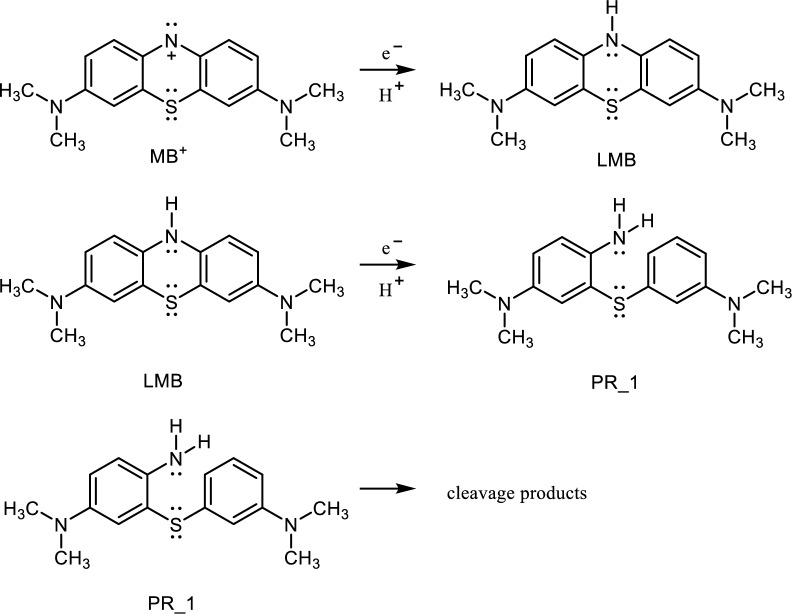
Suggested reductive degradation sequence.

Based on the simulation, a sequence of reactions was proposed in Scheme 2.

Comparison of MB degradation efficiency in the reduction and oxidation processes indicates the important role of the reaction with the electron. In the oxidation process, oxygen reacts with an electron to form a superoxide anion radical, which is involved in further transformations of the dye. The efficiency of this process is lower than that of the reduction process (Fig. 6a), which suggests that the addition of an electron destabilises the chromophore ring and leads to its rupture.

**Scheme 3:**
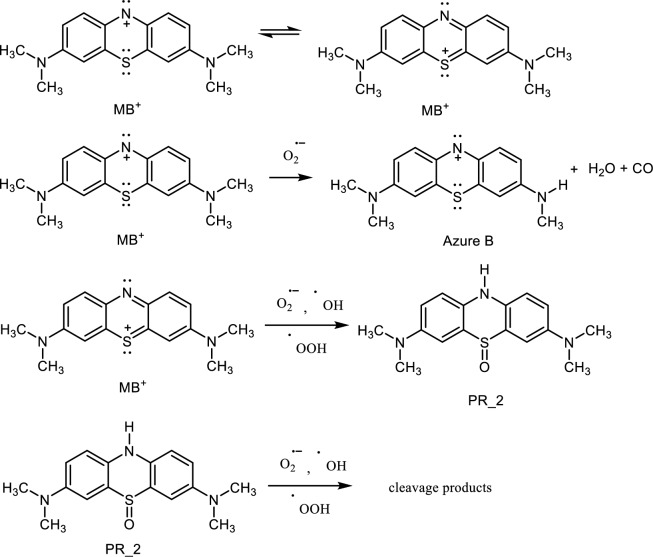
Suggested oxidative degradation sequence.

Regarding the degradation of dyes (especially MB) several articles have been published. Methylene blue is a widely used model compound because of its high water solubility and a convenient way of analysis using UV–Vis spectroscopy measurements. However, few articles on MB degradation on MoS_2_ nanoparticles contain kinetic data. For comparison with the data we obtained, the works of Sahoo et al.^34^ seem to be suitable. They studied MB photocatalytic degradation on MoS_2_ nanosheets of a few layers under visible light. The MoS_2_ catalyst was prepared by exfoliation in the sonicating bath. The exfoliated MoS_2_ shows 45.6% degradation efficiency during 1 h. Sahoo et al. in a recent article^35^ investigated MoS_2_ nanoflowers and nanosheets. The materials were synthesised using a hydrothermal method. The source of sulphur for nanoflowers was thioacetamide, whereas the source for nanosheets was potassium sulphide. The synthesised MoS_2_ nanoflowers and nanosheets were used as photocatalysts for the degradation of methylene blue (MB), malachite green (MG) and rhodamine B (RhB) as standard compound under visible light irradiation. The apparent rate constants k_app_ of the kinetic degradation were 10.27 × 10^–3^; 7.51 × 10^–3^; 16.17 × 10^–3^ min^−1^ for MoS_2_ nanoflowers and 7.71 × 10^–3^; 6.53 × 10^–3^; 6.05 × 10–3 min^−1^ for the nanosheet sample in MB, MG and RhB, respectively (Scheme 3).

## Conclusions

The MoS_2_ studied is a direct band-gap semiconductor and provides new opportunities in photocatalysis. The photocatalytic activity of MoS_2_ was evaluated for the degradation of the methylene blue dye. MB degradation was carried out in two systems: (i) with air access, oxidative process, and (ii) with a constant flow of argon in the presence of hydroxyl radical scavenger, a reduction process. Such a set of experiments allowed us to show differences in the course of MB degradation depending on the reaction conditions. The process under reducing conditions was found to be more effective than under oxidative conditions, with the total loss of dye concentration 99% and 80% in the reducing and oxidising processes, respectively. The degradation of MB during the dark process in the presence of oxygen was observed for the first time. The course of this reaction suggests a flow of charge between MoS_2_ and oxygen adsorbed on the surface.

### Supplementary Information

Supplementary Information.

## Data Availability

The datasets used and/or analysed during the current study available from the corresponding author on reasonable request.
