# Supplementary material for: Layered MoS2: effective and environment-friendly nanomaterial for photocatalytic degradation of methylene blue
Source: Sci Rep. 2023 Aug 29;13:14148. doi: 10.1038/s41598-023-41279-y (PMC10465577; doi:10.1038/s41598-023-41279-y)
Supplement: Supplementary file 1 — Supplementary Information. [file 41598_2023_41279_MOESM1_ESM.pdf]

Supplementary Materials for:

Layered MoS_2: effective and environment-friendly nanomaterial for photocatalytic degradation of methylene blue

Joanna Kisała[*, a], Renata Wojnarowska-Nowak[b], Yaroslav Bobitski[e]

[*] corresponding author: jkisala@ur.edu.pl

[a] Institute of Biology, University of Rzeszow, Pigonia 1 Str., 35-310 Rzeszow, Poland.

[b] Institute of Materials Science, College of Natural Sciences, University of Rzeszow, Pigonia 1 Str., 35-959 Rzeszow, Poland

[c] Centre for Microelectronics and Nanotechnology, Institute of Physics, University of Rzeszow, Pigonia 1, 35-959 Rzeszow, Poland

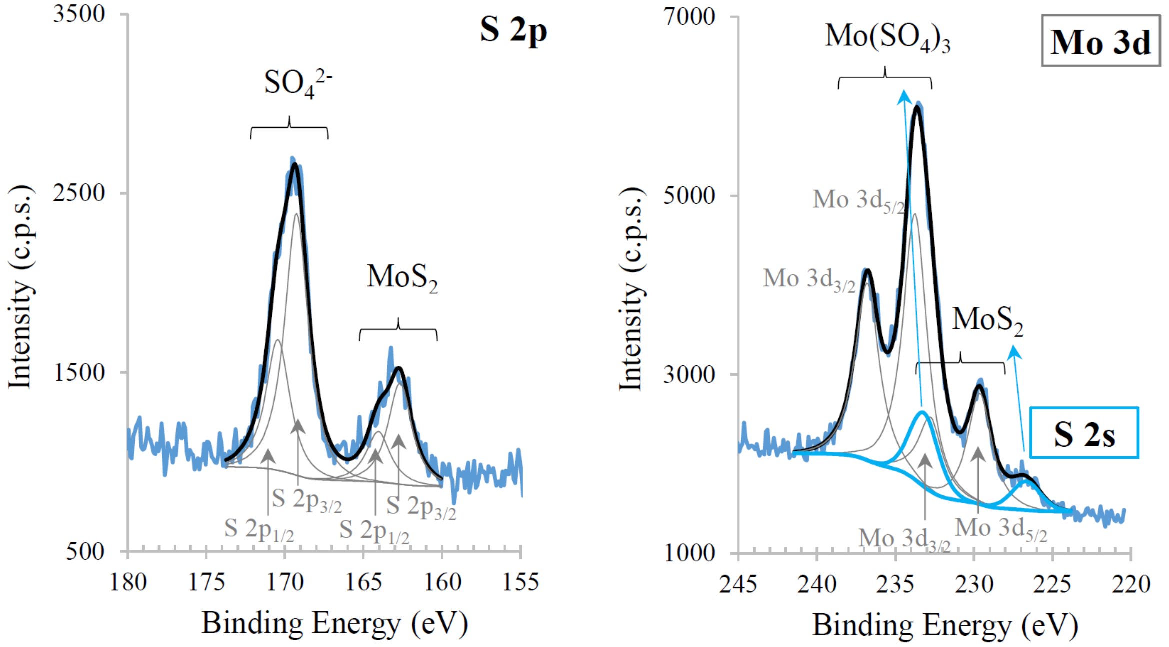

Fig. S1 XPS regions S 2p, Mo 3d and S 2s.

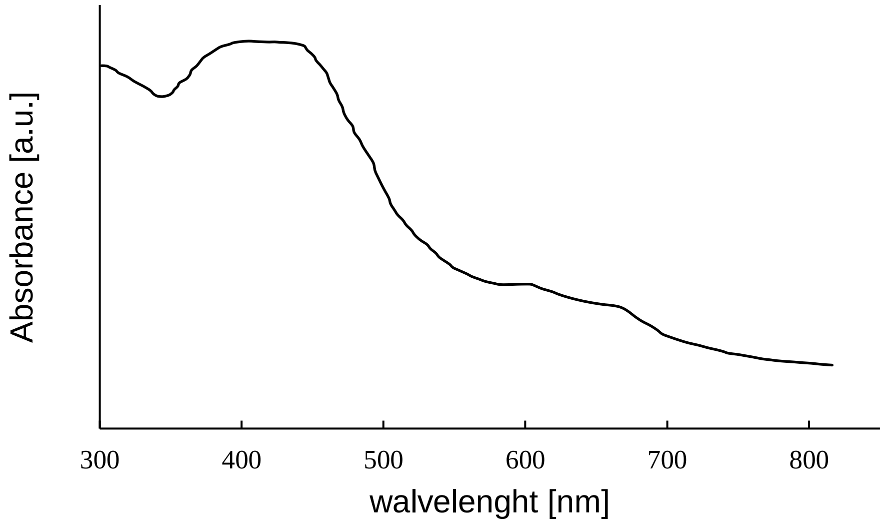

Fig. S2. UV-Vis diffuse reflectance spectra.

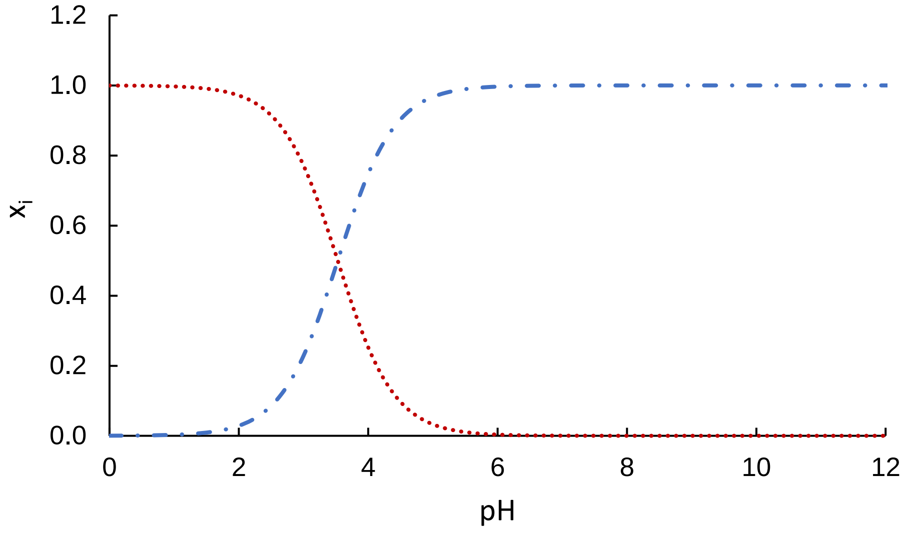

Fig. S3. MB solution ionic species composition depending on pH ($[MB]^{+}$ - blue dot-dash line, $[MBH]^{2+}$ - red dotted line).

Table S1. The rate constants of the radical reactions.

No.	Reaction	Rate constant [$dm^3\ mol^{-1}\ s^{-1}$]
1.	$2\ e_{aq}^{-} \rightarrow H_2 + 2\ OH^-$	1.1×10^{10}
2.	$e_{aq}^{-} + H^{\bullet} \rightarrow H_2 + OH^-$	2.5×10^{10}
3.	$e_{aq}^{-} + {}^{\bullet}OH \rightarrow OH^-$	2.5×10^{10}
4.	$e_{aq}^{-} + H^+ \rightarrow H^{\bullet}$	2.3×10^{10}
5.	$2\ H^{\bullet} \rightarrow H_2$	1.55×10^{10}
6.	${}^{\bullet}OH$ + tert-Butanol $\rightarrow H_2O$	6×10^{8}
7.	$H^{\bullet}$ + tert-Butanol $\rightarrow H_2O$	1×10^{5}
8.	$H^{\bullet} + {}^{\bullet}OH \rightarrow H_2O$	7.0×10^{9}
9.	$H^{\bullet} + H_2O \rightarrow H_2 + {}^{\bullet}OH$	10
10.	$H^{\bullet} + OH^- \rightarrow e_{aq}^- + H_2O$	2.2×10^{7}
11.	$e_{aq}^- + MB^+ \rightarrow MB^{\bullet}$	2.0×10^{10}
12.	$H^+ + MB^{\bullet} \rightarrow LMB^{+\bullet}$	2.0×10^{10}
13.	$H^{\bullet} + MB^+ \rightarrow LMB^{+\bullet}$	1.2×10^{10}
14.	$H^{\bullet} + MB^+ \rightarrow R^{\bullet}$	1.1×10^{9}
15.	$2LMB \rightarrow MB^+ + LMB$	2.0×10^{9}

References

[1] Buxton GV, Greenstock CL, Helman WP, Ross .B (1988) J. Phys. Chem. Ref. Data 17 513-886. https://doi.org/10.1063/1.555805

[2] Elliot AJ (1989) Radiat. Phys. Chem. 34 753-758. https://doi.org/10.1016/1359-0197(89)90279-8

[3] Solar S, Solar W, Getoff N (1982) Z. Naturforsch. 37a 78-85.
